# Does Pathogen Spillover from Commercially Reared Bumble Bees Threaten Wild Pollinators?

**DOI:** 10.1371/journal.pone.0002771

**Published:** 2008-07-23

**Authors:** Michael C. Otterstatter, James D. Thomson

**Affiliations:** Department of Ecology and Evolutionary Biology, University of Toronto, Toronto, Ontario, Canada; University of Utah, United States of America

## Abstract

The conservation of insect pollinators is drawing attention because of reported declines in bee species and the ‘ecosystem services’ they provide. This issue has been brought to a head by recent devastating losses of honey bees throughout North America (so called, ‘Colony Collapse Disorder’); yet, we still have little understanding of the cause(s) of bee declines. Wild bumble bees (*Bombus* spp.) have also suffered serious declines and circumstantial evidence suggests that pathogen ‘spillover’ from commercially reared bumble bees, which are used extensively to pollinate greenhouse crops, is a possible cause. We constructed a spatially explicit model of pathogen spillover in bumble bees and, using laboratory experiments and the literature, estimated parameter values for the spillover of *Crithidia bombi*, a destructive pathogen commonly found in commercial *Bombus*. We also monitored wild bumble bee populations near greenhouses for evidence of pathogen spillover, and compared the fit of our model to patterns of *C. bombi* infection observed in the field. Our model predicts that, during the first three months of spillover, transmission from commercial hives would infect up to 20% of wild bumble bees within 2 km of the greenhouse. However, a travelling wave of disease is predicted to form suddenly, infecting up to 35–100% of wild *Bombus*, and spread away from the greenhouse at a rate of 2 km/wk. In the field, although we did not observe a large epizootic wave of infection, the prevalences of *C. bombi* near greenhouses were consistent with our model. Indeed, we found that spillover has allowed *C. bombi* to invade several wild bumble bee species near greenhouses. Given the available evidence, it is likely that pathogen spillover from commercial bees is contributing to the ongoing decline of wild *Bombus* in North America. Improved management of domestic bees, for example by reducing their parasite loads and their overlap with wild congeners, could diminish or even eliminate pathogen spillover.

## Introduction

Pathogen outbreaks often occur when anthropogenic change brings wildlife into increased contact with humans and domestic animals [1], [2], [3]. Scientists and laypeople alike pay great attention when these outbreaks involve the emergence or re-emergence of infectious diseases of humans, such as acquired immunodeficiency syndrome (AIDS), severe acute respiratory syndrome (SARS), or H5N1 influenza [2], [4]. In contrast, pathogen outbreaks in wildlife rarely receive due attention, even though disease spread, or ‘spillover’, from heavily infected domestic animals has devastated wild populations [5], [6], [7]. The best-known examples of pathogen spillover involve vertebrate hosts, such as the transmission of rabies and distemper from domestic dogs to wild carnivores in Africa [5], [6]. However, human-mediated declines and extinctions of wild insects are also becoming common [8], [9]. In contrast to diseases of vertebrates, we understand little of the aetiology of insect diseases. As noted by Goulson [10] “…if the introduction of a new pathogen were to lead to an epizootic in native insects, it would almost certainly go unnoticed.”

The conservation of insect pollinators is beginning to draw attention because of reported declines in bee species and the ‘ecosystem services’ they provide [11], [12], [13], [14], [15], [16]. Although habitat loss undoubtedly plays a role in these declines [17], [18], [19], [20], disease is also an important factor [21]. Parasitic mites, for example, destroyed 25–80% of managed honey bee (*Apis mellifera*) colonies, and nearly all feral colonies, in parts of the United States during the mid-1990s [22]. The epidemic of ‘Colony Collapse Disorder’, which, in the last year, destroyed 50–90% of colonies in affected honey bee operations, also appears to be the result of a contagious pathogen [23]. However, wild bumble bees (*Bombus* spp.) are also suffering serious declines throughout North America [24], [25] and the UK [17], [26]. A recent report by the National Academy of Sciences concluded that, in North America, a possible cause of bumble bee declines is pathogen spillover from commercially reared bees [27].

Worldwide, five species of bumble bees are reared commercially for the pollination of at least 20 different crops [28]. The sale of commercial *Bombus* has an estimated value of €55 million annually; crops pollinated by bumble bees have a value of at least €12 billion per year [28]. In North America, greenhouses have used commercial *B. occidentalis* (western species) and *B. impatiens* (eastern species) extensively for the pollination of tomato (*Solanum lycopersicon*) and bell pepper (*Capsicum annuum*) crops [29], [30], [31]. However, pathogen (*Nosema bombi*) outbreaks have apparently decimated commercial *B. occidentalis* [28], [32], resulting in the widespread use of *B. impatiens* throughout North America. The concomitant decline of wild *B. occidentalis* and other closely related species in the subgenus *Bombus* sensu stricto [24] is worrisome, given that that this subgenus suffers from uniquely high levels of parasites [33] that are common in commercial *Bombus* [34] and unusually prevalent near certain industrial greenhouses [35]. The increasing use of commercial bumble bees within and beyond their natural ranges [10], [36], and the abundance of disease in commercial hives [32], [34], [35], [37], may have allowed pathogens to invade wild *Bombus* populations [35], [38], [39].

Infected feral animals may transfer pathogens from domestic to wild populations when they interact with wildlife at shared food sources [2]. In the case of bumble bees, infected commercial bees may escape from greenhouses [40] and forage on a variety of plant species shared by wild *Bombus* [41]. At least one pathogen, the intestinal protozoan *Crithidia bombi* (Kinetoplastida, Trypanosomatidae) [42], [43], is known to spread horizontally when infected and susceptible bumble bees share flowers [44]. Infection by *C. bombi* can severely reduce the colony-founding success of queens [45], the fitness of established colonies [45], and the survival [46] and the foraging efficiency [47], [48], [49] of workers. In Europe, *C. bombi* is a well known enemy of bumble bees [50], whereas in North America, almost nothing is known about its occurrence. During the early 1970s, Macfarlane [51] and Liu [52] documented an unidentified flagellate infecting a small proportion (<2%) of Canadian *Bombus*; this parasite was later identified as *C. bombi* (R.P. Macfarlane, pers. comm.). Given that commercial bumble bees were not used in Canada until the 1990s [28], it does not appear that greenhouses were responsible for the first introduction of this pathogen into North America. Nevertheless, *C. bombi* has since become the most prevalent pathogen of commercially reared *Bombus* in Canada [35].

The potential spread of the pathogen *C. bombi* from commercial to wild bumble bees presents a rare opportunity to investigate the dynamics of an emerging infectious disease of wildlife. We constructed a spatially explicit model to explore pathogen spread from a point source into a homogeneous wild bee population, as if by infected commercial bees escaped from greenhouses. We then estimated parameter values for our model using laboratory experiments and the literature on bumble bees and *C. bombi*. Finally, we examined wild bee populations near greenhouses for evidence of pathogen spillover, and compared the fit of our model to patterns of *C. bombi* infection observed in the field. Our results show that spillover of *C. bombi* from commercial bumble bees is both expected and observed near industrial greenhouses. Due to spillover from commercial bees, *C. bombi* is becoming established in wild bumble bee populations and may be contributing to the recent declines of certain *Bombus* species.

## Results

### Predicted spillover of pathogens from commercial to wild bumble bees

In our model (see Materials and Methods), we suppose that infected commercially reared bees that have escaped from a greenhouse deposit short-lived pathogen particles in the environment (e.g., on flowers) near the greenhouse. Susceptible wild bees foraging near the greenhouse acquire infection from these particles and become infectious themselves, i.e., they deposit new infective particles in the environment. Wild bees and pathogen particles (which we imagine can be picked up and carried on bees' bodies) move about the environment via simple diffusion. Using laboratory experiments and the literature, we parameterized our model for the particular case of *C. bombi* infections spreading from commercial to wild bumble bees.

**Figure 1:**
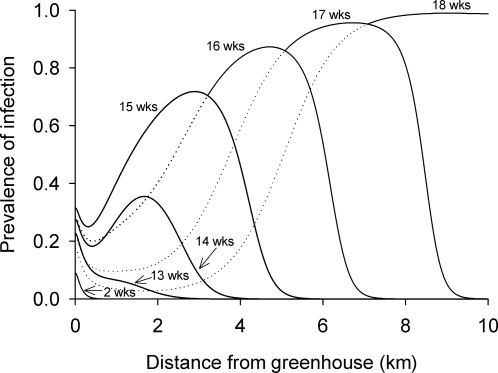
Predicted long-term dynamics of pathogen spillover into wild bumble bee populations near greenhouses. Prevalence curves were generated through numerical simulation of our diffusion model [equations (1)–(3)] using the parameter estimates given in Table 2. This figure illustrates a slow build-up of pathogenic *C. bombi* in the wild population (*t* = 2 wks to *t* = 13 wks; traces for weeks 3–12 omitted for clarity) followed by a large, rapidly forming, wave front of infection (*t* = 13 wks to *t* = 18 wks) that travels away from the greenhouse at approximately 2 km per week.

Figure 1 shows the long-term dynamics of *C. bombi* spillover as predicted by our model. Initially (*t* = 0–13 wks), pathogen spillover into wild populations is localized around the source; the predicted prevalence of *C. bombi* peaks at approximately 20% alongside greenhouses, and declines to 0% at a distance of roughly 2 km. Subsequently, a large wave of infection develops rapidly; between *t* = 14 and 15 wks, peak prevalence of *C. bombi* near greenhouses increases from roughly 35% to 75%. By *t* = 18 wks, peak prevalence reaches ∼100%, and the wave spreads through the wild bumble bee population at a rate of ∼2 km/wk. Recall that our model considers only horizontal transmission of disease among foragers, and not vertical transmission within hives. In nature, pathogens might spread rapidly among nestmates allowing *C. bombi* to establish in wild bee populations sooner than predicted in Figure 1.

We used our model to explore how various aspects of host-pathogen ecology might influence disease spread in a wild bumble bee population. We find that pathogen spillover depends most crucially on the dynamics of transmission at flowers (or, more generally, wherever transmission from commercial to wild bees takes place). For example, halving the estimated rate at which *C. bombi* breaks down on flowers (Figure S1A), or doubling the rate of *C. bombi* deposition on flowers (Figure S1B) or transmission from flowers (Figure S1C), causes the late-season wave of infection to increase by 4–5 times (from ∼20% to ∼90%). This also implies that, all else being equal, pathogen species that remain in the environment (decay slowly) as durable spores could spread extensively if introduced into wild populations. In our model, infection occurs at a rate proportional to the product of the densities of pathogens and hosts; thus, it is not surprising that increases in the net growth rate of the susceptible population has a strong positive effect on pathogen prevalence near greenhouses (Figure S1D). In contrast, a five order-of-magnitude change in the diffusion rate of hosts and pathogens increases the peak prevalence of infection by, at most, ∼30% (Figure S1E). These sensitivity analyses indicate that for each of our model parameters there is a threshold value below which no wave of infection is predicted during late summer, but above which a wave front will form and travel through the wild host population.

### Dispersal of commercially reared bumble bees from greenhouses

**Figure 2:**
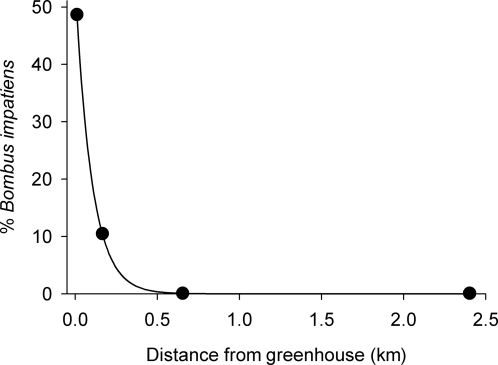
Prevalence of commercially reared bumble bees foraging near an industrial greenhouse. Relative abundance (% total catch of bumble bees, all species) of *Bombus impatiens* workers collected near a greenhouse in southern Ontario during June 2005. Solid line indicates the exponential fit, y = 53.77e^−0.01x^. Most, if not all, of these *B. impatiens* workers were from commercial colonies in the greenhouse (see text).

Pathogen spillover as envisioned in our model requires that infected commercial bumble bees escape from greenhouses and contaminate the local environment with infectious particles. Several lines of evidence support this assumption. First, during each collecting date at our Exeter and Leamington sites (where greenhouses were actively using commercial *B. impatiens* for pollination), we observed *B. impatiens* workers entering and leaving greenhouses through the numerous large vents that are used for temperature control. Many of these bees returned to the greenhouse with visible pollen loads, indicating that they were foraging on wild flowers nearby. Correspondingly, the abundance of *B. impatiens* workers on wildflowers declined with increasing distance from greenhouses. Figure 2 shows that, during early summer (June) at Exeter, almost all (>90%) of the *B. impatiens* were collected within 200 m of the greenhouse, whereas only 1% foraged beyond 400 m despite suitable flower patches at greater distances. The same pattern was evident at our Leamington site. Indeed, half of the bumble bees we collected from wild flowers at both Exeter and Leamington during early summer were *B. impatiens* (for each site, across all collecting distances from greenhouses), yet this species comprised, at the same time of year, only 15% of bumble bees at Beamsville, where greenhouses had stopped using *B. impatiens*, and a third of bumble bees at our site away from any greenhouse operation (Thamesville) (significant differences in the proportion of *B. impatiens* among sites: *G* = 16.3, d.f. = 3, *P* = 0.001). It should be noted that, within the native range of *B. impatiens* (which includes our study sites), it is difficult to say whether a ‘wild-caught’ individual of this species is truly from a wild hive, or if it originates from a commercial hive inside a greenhouse. However, in a preliminary study conducted *outside* of the native range of *B. impatiens* (British Columbia, Canada), we found numerous workers of this species (17 collected per hour) on wildflowers near two industrial greenhouses that use commercial hives for pollination (unpublished data), suggesting that commercial bumble bees are indeed escaping and foraging outside greenhouses. Lastly, it is noteworthy that male *B. impatiens* were also unusually common near greenhouses. At Exeter and Leamington, we caught 27 of these males during June even though wild colonies were just starting to produce workers at this time; indeed, *B. impatiens* males are not normally observed in our study area until at least the end of July (M.C. Otterstatter, unpublished). Hence, it is probable that many of the worker and male *B. impatiens* we observed near greenhouses originated from mature commercial colonies used for pollination.

Although we cannot prove that the commercial bees we observed escaping from Exeter and Leamington greenhouses during summer 2005 were from infected hives, 89% of the colonies (n = 65) that we received from their supplier during 2004–2006 contained the pathogen *C. bombi*, and 73±26% (mean±SD) of nestmates were infected within hives that tested positive for this pathogen. The commercial rearing facility selected these hives from stock destined for industrial greenhouses; hence, these colonies were representative of those used by the greenhouses in our study area.

### Observed spillover of pathogens from commercial to wild bumble bees

In order to test the predictions of our model, we investigated the prevalence of the pathogen *C. bombi* among bumble bees at varying distances to three industrial-scale greenhouse operations. At our two field sites where greenhouses were actively using commercial bumble bees, *C. bombi* infected, on average, 15% (Exeter, n = 273, 4/8 species infected) and 23% (Leamington, n = 129, 3/6 species infected) of foraging workers. Near an industrial greenhouse that had stopped using commercial bumble bees (Beamsville), and away from greenhouses of any kind (Thamesville), wild *Bombus* were entirely free of *C. bombi* (site effect, *G* = 26.9, d.f. = 3, *P*<0.001). We also found *C. bombi* in 10% (n = 20, 2/6 species) of queens and 2% (n = 119, 2/7 species) of male bees caught near greenhouses (sex/caste effect, *G* = 26.0, d.f. = 2, *P*<0.001). Importantly, because our samples do not account for bees that ceased foraging or perished due to illness, we probably underestimate the true prevalence of disease in wild bumble bees.

**Figure 3:**
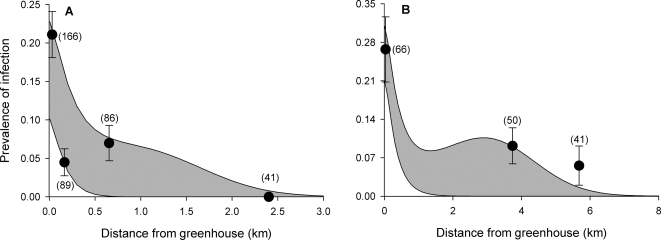
Spillover of pathogenic *Crithidia bombi* into wild bumble bee populations near greenhouses in southern Ontario. Filled circles indicate the observed mean±SE prevalences of *C. bombi* among bumble bee workers (across species and sampling dates) collected at varying distances to industrial greenhouses at (A) Exeter and (B) Leamington during summer 2005. Sample sizes are shown in parentheses. Shaded areas indicate the predicted *C. bombi* prevalences during our study period, based on the diffusion model [equations (1)–(3)] and the parameter values shown in Table 2. In panel A, for example, the lower curve of the shaded area represents the predicted prevalence of infection during our first collecting effort at Exeter, as a function of distance from the greenhouse, whereas the upper curve represents the predicted prevalence during our last collecting effort, nine weeks later. We estimated that, in our numerical simulations, *t* = 4–13 wks (Exeter) and *t* = 5–14 wks (Leamington) most closely matched with our nine week sampling period during June–August (see Materials and Methods).

The prevalence and intensity of *C. bombi* infections in bumble bees declined with increasing distance from greenhouse operations (Figure 3; Table 1). Up to 33% (Exeter) and 47% (Leamington) of bees collected immediately adjacent (within 30 m) to greenhouse operations were infected; however, no infected bees were found at 2.4 km from the greenhouse at Exeter, and only 5% were infected between 5–6 km from the greenhouses at Leamington (distance×site interaction, Table 1). A second pathogen, the microsporidian *Nosema bombi*, occurred only at Leamington, and only in *Bombus fervidus*; nevertheless, its prevalence also declined with distance from greenhouses (31% of bees infected within 30 m, 25% at 3.7 km, 0% at 5.3 km and beyond; Cochran-Armitage test for trend, *Z* = 2.3, *P* = 0.01, n = 40). Importantly, the prevalence of *C. bombi* declined with increasing distance from greenhouses among all *Bombus* species (host species×distance: Exeter, *G* = 0.4, d.f. = 3, *P* = 0.55; Leamington, *G* = 1.9, d.f. = 3, *P* = 0.17) and this decline remained significant even when we excluded *B. impatiens* from the analysis (*G* = 4.2, d.f. = 2, *P* = 0.04). Thus, this effect was not simply due to us catching fewer infected commercially reared *B. impatiens* as we moved away from their hives inside greenhouses: greenhouses were foci of infection for all bumble bees.

**Table 1:** Statistics comparing the prevalence of *C. bombi* infections in bumble bees (all species pooled) across study sites, times of year, and distance from industrial greenhouse operations.

Explanatory variable*	*G*	d.f.	*P*
Study site^a^	2.4	1	0.12
Time of year^a^	3.7	1	0.06
Distance from greenhouse^b^	8.9	1	0.003
Site×season	10.7	1	0.001
Distance×site	11.0	1	0.001

a ‘Study site’ (Exeter or Leamington) and ‘Time of year’ (early [June] or late [July, August]) treated as nominal variables.

b ‘Distance’ treated as a continuous variable.

* Non-significant interactions are not shown.

Bees foraging immediately adjacent to greenhouses also harboured significantly more intense infections, i.e., they carried more pathogen cells in their gut tracts, than bees collected further away (*Z* = −2.0, *P* = 0.04, n = 67). Infection intensity did not differ between our two study sites (*Z* = −0.71, *P* = 0.48, n = 67) or among host species (χ^2^ = 4.4, *P* = 0.36, n = 67). Given that our collecting locations at each site had similar compositions of bee species during most of the summer (mid- to late-summer sampling dates: Exeter, *G* = 6.3; d.f. = 3, *P* = 0.18; Leamington, *G* = 2.0, d.f. = 3, *P* = 0.37), and that we sampled concurrently at varying distances from greenhouses, the observed patterns in pathogen prevalence and intensity are probably not the result of seasonal changes in pathogen abundance or heterogeneities in the host population.

Based on our parameter estimates (Table 2), the spillover model provided a good fit to the pathogen prevalences that we observed in the field. Figure 3 shows that our model reproduced the sharp decline in pathogen prevalence observed near greenhouses and matched well with the prevalences that we observed over several kilometres away. The model predicts that, for nearly any given distance from a greenhouse operation, the prevalence of infection would vary by less than 10% between June–August. Although our field study cannot be considered a rigorous test of the model, it is encouraging that the observed average prevalences of infection typically fell within this narrow predicted range. More intense sampling (e.g., every few days) of wild bee populations is needed to determine if our model accurately predicts epizootic waves and week-to-week changes in pathogen prevalence near greenhouses.

**Table 2:** Parameter estimates for our model of *Crithidia bombi* spillover near greenhouses.

Parameter	Symbol	Value
Birth rate of the susceptible population	*a*	0.220 d^−1^
Natural (non-disease) mortality rate	*b*	0.183 d^−1^
Disease-induced mortality rate	*α*	0.102 d^−1^
Pathogen production rate	*λ*	4.23×10^4^ d^−1^
Pathogen decay rate	*μ*	12.98 d^−1^
Transmission rate	*ν*	1.08×10^−4^ m^2^ d^−1^
Initial host population density^a^	*S*_0_	0.08 m^−1^
Diffusion coefficient	*D*	8000 m^2^ d^−1^

a Initial host density based on data in Forup and Memmott [83].

Aside from *B. impatiens*, workers of two other bumble bee species were frequently infected by *C. bombi* near greenhouses: *B. rufocinctus* at Exeter and *B. bimaculatus* at Leamington (site×species effect, *G* = 11.3, d.f. = 3, *P* = 0.010; Table 3). Although it is impossible to distinguish commercial *B. impatiens* from their wild counterparts, these other two species are not produced commercially and must therefore have come from wild colonies. Interestingly, our analysis of plant species use by bumble bees (Table 4) shows that the wild species that often shared flowers with commercial *B. impatiens* (e.g., *B. rufocinctus*) were more often infected by *C. bombi* than those species that rarely shared flowers with *B. impatiens*. A simple correlation analysis revealed a significant positive association (Pearson's rho = 0.8, n = 5, *P* = 0.04) between percent similarity in plant species use (Table 4) and prevalence of infection (Table 3).

**Table 3:** Average (±SE) percentage of bumble bees (sexes and castes pooled) infected by *Crithidia bombi* among the most common *Bombus* species at our study sites in southern Ontario during June–August, 2005.

Species	Site	
	Exeter	Leamington
*B. bimaculatus*	1.52±0.96 (66)	20.00±6.33 (30)
*B. fervidus*	1.45±0.92 (69)	5.71±2.87 (35)
*B. griseocollis*	0.00±0.00 (17)	0.00±0.00 (3)
*B. impatiens*	17.46±2.59 (189)	26.83±4.60 (82)
*B. rufocinctus*	45.45±10.26 (22)	–
Other	0.00±0.00 (38)	0.00±0.00 (6)

**Table 4:** Percent similarity in plant species use among bumble bee species at our study sites in southern Ontario during June–August, 2005 (values for Exeter shown below the diagonal, those for Leamington shown above the diagonal).

Species^a^	*B. impatiens*	*B. rufocinctus*	*B. bimaculatus*	*B. fervidus*
*B. impatiens*	100	-	8.8	12.6
*B. rufocinctus*	44.8	100	-	-
*B. bimaculatus*	20.4	31.6	100	18.8
*B. fervidus*	8.6	10.0	12.9	100

a rarely collected *Bombus* species (n<15 per site) are not shown; also, note that no *B. rufocinctus* were collected at Leamington.

## Discussion

Introduced pathogens often spread rapidly and devastate naïve host populations. Among wildlife, diseases may be introduced via the spread, or ‘spillover’, of pathogens from heavily infected domestic animals [5], [6]. Here, we use a combination of mathematical modelling and field data to show that spillover from commercially reared bumble bees has introduced the contagious pathogen *Crithidia bombi* into wild bumble bee populations. During two years, and across nine sites in southern Ontario including our previous work: [35], we have found *C. bombi* infecting up to 75% of wild bumble bees, depending on the time of year and the host species, near industrial greenhouses that use commercial *Bombus* for pollination. At sites distant to greenhouses, we have not found any bees harbouring this pathogen. Furthermore, we show that the prevalence and intensity of *C. bombi* infections decline with increasing distance from greenhouses. Given that wild bumble bee populations in our area were almost entirely free of *C. bombi* [average prevalence = 1.5%], 52 before to the use of commercial *Bombus* in Canada [*ca*. 1990, 28], our results suggest a dramatic increase in infection rates near greenhouses.

Pathogen prevalences near greenhouses are generally consistent with our model of spillover, which predicts *C. bombi* invasion of wild bee populations under a range of assumptions about the dynamics of transmission. In the field, we see clear evidence of the early stages of spillover, with frequent primary infections (from commercial to wild bees) near greenhouses. However, we did not observe a large wave of secondary infections among wild bees as predicted by our model and which might be expected based on previous studies of insect diseases [53], [54], [55]. There are at least two explanations for this discrepancy. First, our model does not predict an obvious wave of infection until 14 wks after spillover first occurs (i.e., when commercial and wild bees first interact), which would be after wild bumble bees have completed their colony cycle in our area and after greenhouses have stopped using commercial colonies for the season. In warmer regions, such as Central and South America (where commercially reared *Bombus* are becoming increasingly common), wild bumble bees emerge earlier in the year or remain active year-round; in these areas, wild and commercial species may overlap for a lengthy period and, under such conditions, our model predicts massive spillover into wild populations. Nevertheless, seasonal forcing in temperate regions does not mean there is no lasting impact of spillover on wild bee populations. New queens, emerging in the fall from colonies near greenhouses, may acquire *C. bombi* from their infected natal hives or from contaminated flowers. Such infections can harm queens during protracted winter hibernation (via accelerated loss of body mass) and reduce or eliminate their ability to found a new colony in the spring [45]. Those few infected queens that manage to establish a new hive will have smaller, less productive, colonies than uninfected queens [45]. The second reason why we may not observe epizootic waves is that such waves are predicted to move very rapidly and, in their wake, leave few wild bees and only low prevalences of *C. bombi* near greenhouses (see, for example, week 18 in Figure 1). Future studies should sample wild bumble bee populations on a weekly basis near greenhouses (or other agricultural operations that use commercial *Bombus*) to help identify travelling waves of infection. Areas where commercial and wild bees overlap for several months deserve the greatest attention. Our model is only a first step in understanding the dynamics of pathogen spillover in this system; further study of disease transmission at flowers, for example, is clearly needed.

More broadly, the spillover of pathogens from commercial to wild bumble bees is an example of human-mediated pathogen invasion, which has been implicated in wildlife declines and extinction events over the past 40,000 years [6], [56], [57]. Historically, the development of agriculture resulted in large populations of domestic animals, which facilitated the build up and transmission of disease among wild and domestic animals and humans [4], [58]. International trafficking of domestic animals has also contributed to pathogen emergence and spillover [59]. Similarly, bumble bee domestication (bombiculture) has produced dense monocultures of hives within rearing facilities and greenhouses; under these conditions, contagious disease has flourished [28], [32], [34], [37]. Given the worldwide expansion of bombiculture, it is imperative that commercial rearing facilities work to achieve and maintain disease free bumble bees for crop pollination.

Recent devastating losses of honey bees due to ‘Colony Collapse Disorder’, which appears to be the result of a virus introduced from Australia [23], has brought much attention to the issue of pollinator health. Unfortunately, it is still not widely recognized that wild populations of many native bees are also in danger of collapse. In North America, certain *Bombus* species have experienced recent precipitous declines [25]. Although widespread, these declines seem restricted primarily to species in the subgenus *Bombus* sensu stricto, particularly *B. affinis*, *B. franklini*, and *B. occidentalis* [24], [60]. It is noteworthy that this subgenus is especially susceptible to parasitic infection [33], and that its decline coincided with a devastating parasite epidemic among commercially reared, congeneric, *B. occidentalis* [61]. In the lab, pathogen spread occurs most easily among closely related bumble bees [44], [62], [63]; thus, any pathogens that escaped from infested commercial *B. occidentalis* would most likely have spread to wild *Bombus* sensu stricto. Based on our model, and our observations near greenhouses, it is probable that destructive pathogens have been spilling over into wild bee populations since the collapse of commercial *B. occidentalis* during the late 1990s, and this has contributed to the ongoing collapse of wild *Bombus* sensu stricto.

In the case of bumble bees, the mechanism of pathogen spillover is clear: infected commercial bees frequently escape from greenhouses and share nearby flowers with wild *Bombus*, thereby providing sufficient opportunities for the transmission of disease. We often found escaped commercial bees on flowers near greenhouses, which is consistent with prior observations [bumble bees: 40,41; honeybees: 64]. Previous work shows that *C. bombi* is present in the nectar of wild flowers in Europe (where this pathogen is common among wild *Bombus*), and that shared flower use by healthy and infected bees results in transmission [44]. It is noteworthy that prevalences of *C. bombi* in our study reflected patterns of plant species use by the hosts. *Bombus* species that overlapped heavily with commercial *B. impatiens* at flowers experienced higher rates of infection than species that seldom shared plants with commercial bees. Although we cannot demonstrate a firm link between plant species use and infection risk, such a pattern is expected simply through the non-random visitation, and contamination, of plant species by infected bees [65]. It is also possible that spillover occurs via wild bees entering greenhouses and visiting contaminated plants/bee hives, or via infected commercial bees visiting wild colonies outside the greenhouse [‘drifting’, 66]. Regardless of the mechanism(s), spillover would be reduced, or perhaps even eliminated, if greenhouses were modified to prevent the cross-traffic of commercial and wild bees. Simple mesh screens, fitted to the ventilation systems of greenhouses, would minimize both the loss of costly commercial pollinators and the entrance of wild species [41], [64].

## Materials and Methods

### A model of pathogen spillover

We based our model of pathogen spillover on the standard insect-pathogen model of Anderson and May [67], with the addition that we track the spread of pathogens in space *x* (displacement from starting point) as well as time *t*:
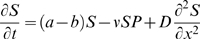
(1)

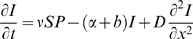
(2)

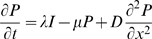
(3)where *S*, *I*, and *P* are the densities of susceptible wild bees, infected wild bees, and infective pathogen particles in the environment, respectively, *a* is the birth rate of the susceptible population, *b* is the natural (non-disease) mortality rate, *ν* is the transmission rate of pathogen particles, *α* is the disease-induced mortality rate of infected bees, *λ* is the rate at which infected bees produce and deposit pathogen particles in the environment, *μ* is the ‘decay’ rate at which pathogen particles breakdown in the environment and become uninfective, and *D* is the dispersal rate of hosts and pathogen particles.

Our model considers the within-season dynamics of disease only and includes the following noteworthy simplifying assumptions: first, hosts remove a negligible amount of pathogen particles from the environment relative to the amounts that are produced and decay; second, no terms are included to capture the dynamics of infection within colonies (e.g., vertical transmission) or during the solitary phase of queens (e.g., during hibernation). We simplified the model in this way because we wish to focus on the introduction of pathogens by commercial bees into an established population of wild bumble bees, and the subsequent horizontal transmission of infection among foraging workers. The first assumption is justified by our parameter estimates (see below), which show that λ and μ are several orders of magnitude larger than ν [67]. The second assumption was made in order to minimize the number of unknown and currently inestimable parameters in our model. Nevertheless, we point out that vertical transmission might be an important aspect of pathogen spillover, particularly during early summer when bee colonies are small and vulnerable to disease-induced mortality. We ignored the infection of queens because, at least during the summer, only about 5% of bees infected by *C. bombi* are sexuals, suggesting that almost all transmission occurs among workers.

Equations (1)–(3) constitute a reaction-diffusion model, which describes the ‘reaction kinetics’ between pathogens and hosts plus their diffusive movement through the environment. Initially (at time *t* = 0), pathogens are introduced (at spatial location *x* = 0) into a uniformly distributed bee population. We imposed ‘no flux’ boundary conditions, i.e., the rate of change in the densities of hosts and pathogens is zero at the edges of space (*x* = 0 and 10 km from the starting point). Our model considers only one spatial dimension, which is appropriate for pathogen spread from a point source (as is the case in our study system) [54], [68]. By reducing the spatial dynamics to a single dimension, the model assumes that pathogen spread away from the point source is the same in all directions. The diffusion terms assume that hosts and pathogen particles move randomly in all directions [69], which is typical of insect-pathogen models [54], [55], [70], [71], [72].

In our study system, infected bumble bees leave pathogen cells at flowers (perhaps by defecating while on or near plants, or by carrying infective cells on the outer surfaces of their bodies) and these cells may be picked up by subsequent visitors [44] and dispersed to other flowers. Thus, although these ‘free-living’ pathogen cells do not diffuse appreciably under their own power, we assume that hosts carry and disperse them throughout the environment e.g., [73]; other flower-visiting insects might also disperse bee pathogens in the same way e.g., [65], [74]. Hence, our dispersal coefficient *D* has the same value for hosts and free-living pathogen cells (i.e., pathogen cells disperse to the same extent as the hosts that carry them). The validity of assuming random, diffusive movement of pathogen cells is supported by the fact that bumble bee populations isotropically disperse other pathogenic particles (e.g., anther smut) among plants over relatively short distances [75], [76]. Furthermore, simple diffusion is sufficient to capture the dynamics of other insect-pathogen interactions [77]. Although our diffusion model may oversimplify the intricate movements of foraging bees, it serves as a useful foundation on to which one can add more complicated mechanisms of dispersal e.g., [69].

### Estimating model parameters

We wished to determine if our model of pathogen spread, once parameterized with known information about the behaviour of bumble bees and their pathogens, could be used to predict patterns of disease near commercial greenhouses. Therefore, we conducted small-scale laboratory experiments to estimate two critical parameters in the transmission process, the rates of pathogen production, *λ*, and decay, *μ*; the methods and results of these experiments are presented in Supplementary Text S1 and Figure S2. We estimated the remaining parameters from the literature; further details are presented in Supplementary Text S2.

### Spatial spread of pathogens in the field

Table S1 summarizes our four study sites in south-western Ontario. Sites were surrounded by agricultural fields and had similar plant and bumble bee species. At three sites, we collected bees near a large (>15 acre) greenhouse operation that used commercial bumble bees for pollination of tomatoes or bell peppers; at two of these sites, we also sampled throughout the summer along transects running away from the greenhouse. Our fourth site, for comparison, had no greenhouses within 50 km. We mapped each collecting location to within ±5 m using a Garmin Global Positioning System (GPS). It was not possible to collect bees continuously along our transects because some areas were mowed and devoid of wild flowers; nevertheless, we were able to collect bees closer than (1–2 km) and further than (3–6 km) the modal distance that bumble bees are known to forage from their nests e.g., [78]. All field work was carried out during summer 2005.

At each sampling location, we collected bees during mornings and afternoons by walking haphazard trajectories and catching all visible workers, males, and queens with sweep nets. We held bees in individual plastic vials and transported them to the laboratory in a cooler with ice packs. We identified each individual to species following Laverty and Harder [79]. Using the methods of Colla et al. [35], we examined the gut tracts and fat bodies of bees at 160× magnification and scored their intensity of infection (1 = light infection [≈10–100 cells observed] to 3 = heavy infection [≈1000 or more cells observed]). In total, we collected 468 workers, 123 males, and 24 queens across nine bumble bee species during summer 2005.

We obtained from local growers information on the size and productivity of the greenhouses near our study sites. During our study period, these greenhouses used commercial *Bombus impatiens* Cresson continuously (old colonies are regularly replaced with young ones) from February to June (Beamsville, ∼6 colonies for 18 acres) or February to September (Exeter, ∼300 colonies for 36 acres; Leamington, ∼125 colonies for 35 acres), and had been doing so for about 5–10 years. Based on typical colony sizes[40], the Exeter and Leamington greenhouses probably contained about 18 000 and 7 500 bumble bees, respectively, during the times we sampled these sites. The Beamsville greenhouse, in contrast, had not used bumble bees for approximately two months prior to our sampling.

During 2004–2006, we regularly received *B. impatiens* colonies from a commercial rearing facility that was the sole supplier of greenhouse operations at Exeter and Beamsville, and one of two suppliers of the greenhouses at Leamington. The rearing facility selected our hives from stock destined for industrial greenhouses; thus, these colonies were representative of those used by the greenhouses in our study area. We screened each colony for *C. bombi* by removing 10 arbitrarily chosen workers and examining their gut tracts at 160× magnification. In total, we examined 65 colonies in this manner.

### Data Analysis

We used logistic regression [80] to examine how the probability of *C. bombi* infection at artificially contaminated flowers varied with the size of the inoculum and the delay between inoculation and ingestion. We included bee size (radial cell length) as a covariate in this analysis. Similarly, we examined differences in pathogen prevalence between sites and times of year (nominal explanatory factors), and with distance from industrial greenhouses (continuous explanatory factor) using logistic regression. We also used this analysis within a site to compare prevalence among host sexes/castes (queens, workers, males), and host species. We pooled rarely collected (n<20 for all sites) species for these analyses. In all cases, we used the infection status (yes/no) of each bee as our binary dependent variable. The test statistic for the logistic regression is the likelihood ratio (*G*). We compared our intensity of infection scores using the non-parametric Wilcoxon two-sample test (between sites) and the Kruskal-Wallis test (between host species) [81]. We examined the similarity among bumble bee species (workers only), in terms of the plant species they visited, by calculating percent similarity [82] from the numbers of individuals of each bee species collected from each plant species at a site. We wished to determine if bee species that often shared flowers with commercial *B. impatiens* experienced greater prevalences of infection by *C. bombi* than species that rarely shared flowers with commercial bees; thus, we restricted this analysis to include only collecting sites immediately adjacent to greenhouses (where the vast majority of commercial *B. impatiens* were found, see Results) and only mid- to late-summer sampling dates (when *C. bombi* infections were most common, see Results). As a result, this analysis included only *B. bimaculatus*, *B. fervidus*, *B. impatiens*, and *B. rufocinctus*; all other species (e.g., *B. griseocollis*) were too rare to accurately characterize their use of plant species. Similarity values can range from zero (no overlap at any plant species) to 100 (identical use of plant species).

In order to compare the seasonal prevalence of *C. bombi* observed near greenhouses with that expected based on our spillover model, we must match our collecting dates with the appropriate time points in the numerical simulation of equations (1)–(3). However, because we did not observe the spring (May–June) emergence of wild *Bombus* workers at our study sites, the exact date corresponding to *t* = 0 in our simulation, i.e., the beginning of seasonal pathogen spillover from commercial to wild bees, is uncertain. To overcome this difficulty, we used available phenological data for wild bumble bees at nearby sites (M.C. Otterstatter, unpublished), and the observed abundance of wild workers at our study sites during early summer (June), to back-calculate the dates of emergence: roughly four weeks prior to our first collecting date at Exeter, and five weeks prior to our first collecting data at Leamington. A slightly earlier emergence at Leamington than Exeter is consistent with the differing latitudes of these sites. Thus, we estimate that our nine week study period most closely matched with *t* = 4–13 weeks (Exeter) and *t* = 5–14 weeks (Leamington) in our numerical simulation.

## Supporting Information

Text S1 Experimental parameter estimates
(0.06 MB DOC)

Text S2 Parameter estimates from the literature
(0.07 MB DOC)

Figure S1 The sensitivity of our diffusion model to variation in parameter estimates. In each panel, we show how variation in a single parameter affects the predicted prevalence of *C. bombi* during late summer (*t* = 14 weeks in the model), relative to the distance from the source (greenhouse). We explore a range of decay rates (*μ* in d^−1^, panel A), pathogen production rates (*λ* in d^−1^, panel B), transmission rates (*ν* in m^2^ d^−1^, panel C), net rates of increase in the foraging bee population (*r* = *a*−*b* , in d^−1^, panel D), and diffusion rates (*D* in m^2^ d^−1^, panel E). All curves were generated by numerical simulation of equations (1)–(3), and all parameters (except the one of interest in each panel) were fixed according to the values in Table 2. Where possible, we chose biologically reasonable values for our study system (e.g., pathogen production rate); if no such information was available (e.g., transmission rate), we used a range of plausible values that illustrate the behaviour of our model.
(1.14 MB TIF)

Figure S2 Temporal decline in the infectivity of pathogenic *C. bombi* cells deposited at flowers. Each point represents a single bumble bee's (n = 76) predicted probability of infection from the logistic regression including bee size and dose as covariates (see Materials and Methods). The solid line, indicating the time-dependent decrease in infectivity, is a linear regression (y = −0.0038x+0.58, *P*<0.05, *R* ^2^ = 0.59) fitted to the predicted probabilities of infection.
(0.79 MB TIF)

Table S1 (0.04 MB DOC)
