# Supplementary material for: Does Pathogen Spillover from Commercially Reared Bumble Bees Threaten Wild Pollinators?
Source: PLoS One. 2008 Jul 23;3(7):e2771. doi: 10.1371/journal.pone.0002771 (PMC2464710; doi:10.1371/journal.pone.0002771)
Supplement: Text S1 — Experimental parameter estimates (0.06 MB DOC) [file pone.0002771.s001.doc]

## Supplementary Text S1

### Experimental estimates of model parameters

#### Rate of pathogen decay rate at flowers (µ)

We estimated the rate at which infectivity decays by inoculating flowers with *C. bombi* and then experimentally varying the amount of time before susceptible bees visited these flowers. We also varied the number of *C. bombi* placed on flowers in order to estimate how the probability of infection varies with dose. Durrer and Schmid-Hempel [1] showed that transmission at flowers, specifically the contamination of floral nectar, is an important route by which *C. bombi* could spread between host colonies and species. Although *C. bombi* does not possess any durable life stages (e.g., spore, cyst, inclusion body), infectivity (in this case, the probability of infecting a new host) declines only about 10-20% when cells are left exposed in the laboratory for 45 min [2]. Thus, *C. bombi* may remain infective for several hours within the protected nectaries of flowers.

In all experiments, we used *Bombus impatiens* originating from a commercial rearing company in North America. We prepared our inocula by mixing in 30% sucrose the rectal fluids of workers from three colonies infected by *C. bombi*. We estimated the concentration of *C. bombi* using an improved Neubauer haemacytometer (Reichert) and then created two dilutions: 2500 or 250 *C. bombi* per microliter. We obtained healthy bees by transferring brood clumps from several colonies into clean plastic containers and collecting adult workers immediately as they emerged. Newly emerged bumble bees never harbour *C. bombi* (pers. observation), and our protocol prevented healthy bees from contacting (potentially) infected nestmates or food prior to the experiment. Once the adults had reached at least four days of age, each bee was starved for one hour and then allowed to forage individually in a screened cage (91 cm x 41 cm x 62 cm) on fast-cycling *Brassica rapa* plants, which we grew in the laboratory from seed (Wisconsin Fast Plants**)**. Prior to introducing a bee to the flight cage, we added 4 μl of *C. bombi* inoculum (either 10,000 cells [High Dose, n=39] or 1000 cells [Low Dose, n=37], randomly assigned) to the nectaries of a single flower on a single plant. In roughly half of the trials, bees were presented with the inoculated plant immediately (No Delay treatment, n=36); whereas in the remaining trials, we allowed 60 min to elapse between inoculating the flower and allowing the bee to feed from it (Delay treatment, n=40). Thus, our experiment was a factorial design in which Dose and Delay were fully crossed. We conducted our experiments at ~23°C under broad-spectrum fluorescent light. We removed a bee from the flight cage shortly after it had visited the inoculated flower (No Delay treatment: 0–75 min; Delay treatment: 60-100 min). We noted any bees (n=5) that foraged but did not visit the inoculated flower; none of these developed infections. Between bees, we cleaned the flight cage thoroughly with soap and water, and fresh plants were prepared. After foraging, we placed bees in individual vials at 27°C, and fed them a constant supply of 30% sucrose solution. We also immediately inspected each inoculated flower at 12X magnification to ensure that the bee had consumed the contaminated nectar. Ten days later, we sacrificed and screened bees for *C. bombi* using previously established methods [3]. We recorded a bee as infected if any *C. bombi* were found in its gut. We removed an additional 50 ‘Control’ bees from our source population to confirm that *C. bombi* was indeed absent. We housed and screened Control bees for infection in the same manner as the foraging bees; however, none developed infections.

These laboratory experiments showed that, on flowers, the infectivity of the pathogen *C. bombi*, in terms of its probability of infecting a foraging bumble bee, declined quickly over time (*G* = 7.9, d.f. = 1, *P* = 0.005; Figure S2). Roughly half (14/25) of the bees that visited an artificially contaminated flower became infected when the delay between contamination and visitation was less than 10 min. When *C. bombi* remained at flowers for longer than 10 min, infectivity declined steadily until, after 85 min, the probability of infection was less than 15% (1/7). The number of *C. bombi* cells that we placed on a flower (1000 or 10000) did not alter the probability of bees becoming infected (*G* = 0.40, d.f. = 1, *P* = 0.53), and dose interact with delay (*G* = 0.27, d.f. = 1, *P* = 0.60). Probability of infection did not vary with bee size (*G* = 0.51, d.f. = 1, *P* = 0.48), and bee size did not interact with delay (*G* = 2.09, d.f. = 1, *P* = 0.15). Over time, some evaporative loss of inocula may have occurred even within the protected nectaries of our flowers, and this may have contributed to the observed decline in infectivity. However, previous work [1] shows that the infectivity of *C.bombi* declines by only 10-20% even when an inoculum is allowed to dry up almost entirely. It may instead be the case that chemicals naturally present in floral nectar adversely affect the infectivity of *C. bombi* (Manson and Otterstatter, in prep.). The rate of decline observed here corresponds to a half-life of about 77 min for *C. bombi* at flowers and an exponential decay rate *μ* = 12.98 d^-1^. We explore the sensitivity of our model to variation in *μ* in Figure S1A.

#### Rate of pathogen deposition at flowers (λ)

For our model, the ideal measurement of pathogen production would be the rate at which infected bees leave *C. bombi* at flowers. Although other modes of transmission may be possible [e.g., ‘drifting’ by infected workers, 4], contamination of flowers is the primary mode envisioned in our modeling process. We estimated the rate of *C. bombi* deposition on flowers using the simple model of Otterstatter and Thomson [3], which gives the maximum number of *C. bombi* an infected bumble bee might leave at flowers per minute of foraging, as a function of the intensity of infection in its gut. In order to measure the infection intensities in the guts of commercial bees, which are the primary source of *C. bombi* at our study sites, we examined foragers from three commercially reared *B. impatiens* hives.

The hives (initially containing ~30 workers each) originated from the same commercial rearing company that supplies industrial greenhouses in our study area (described below). We connected each hive to a flight cage in the laboratory and gave each worker a unique color mark with typing correction fluid. Bees were allowed to forage freely on artificial ‘flowers’ (arrays of 1.5 mL microcentrifuge tubes, inserted upright into holders made of Styrofoam blocks and stocked with 30% sucrose solution); colonies were supplemented with pollen. Every 2-3 days over the course of several weeks, we randomly removed foragers from the arrays of artificial flowers and quantified the intensity of *C. bombi* in their guts following previously established methods [3]. In total, we screened 45 (n=13, 13, and 19) foragers that were between 7-23 days of age, which is roughly the age range of wild foraging bumble bees [5]. Although our colonies did not experience a natural environment, our lab conditions are probably similar to the controlled environment of an industrial greenhouse.

We found that *C. bombi* infected 89% (40/45) of foragers in the three commercial colonies that we used to estimate the rate of pathogen production/deposition by infected bees. Regardless of colony, bees harboured a similar range of infection intensities: gut infections varied in intensity from 100 – 32,000 cells/μL, with a median value of 2925 cells/μL. Based on the model of Otterstatter and Thomson [3], our median value indicates that a typical infected forager could deposit an estimated maximum of 35235 *C. bombi* cells per minute on flowers. A single bumble bee often spends at least two hours per day foraging [from data in 6,7] and therefore might deposit 4.23 x 10^6^ *C. bombi* per day at flowers. This value assumes that bees defecate on every flower they visit; however, in the lab, we have observed that bees usually defecate only once per foraging bout (~100 flower visits) or less. Thus, it is reasonable to assume that infected bees leave on flowers only about 1% of the cells they might maximally leave, which suggests a value for *λ* of 4.23 x 10^4^ *C. bombi* deposited per day at flowers. Clearly, this calculation involves several rough estimates and assumptions; also, the infection intensities that we observed in our three colonies suggest that pathogen production can vary widely among individuals (probably due to differences in age, body size, and genotype). We therefore examine the sensitivity of our model to changes in *λ* in Figure S1B.

#### Dispersal of bees and pathogens (D)

At our Exeter field site, the density of *B. impatiens* declined exponentially with increasing distance from a greenhouse operation (Figure 2). These bees occurred too early in the summer to be wild *B. impatiens*. Further, we found them only near a greenhouse that was stocked with commercial *B. impatiens* hives; thus, we conclude that they were commercially reared bees foraging from hives within the greenhouse. We used the observed exponential decline as the initial spatial distribution of pathogen particles in our model. This initial condition assumes that the density of pathogens at any point outward from the greenhouse is proportional to the abundance of commercial bees that we observed foraging at that distance.

# References

1. Durrer S, Schmid-Hempel P (1994) Shared use of flowers leads to horizontal pathogen transmission. Proceedings of the Royal Society of London Series B 258: 299-302.

2. Schmid-Hempel P, Puhr K, Kruger N, Reber C, Schmid-Hempel R (1999) Dynamic and genetic consequences of variation in horizontal transmission for a microparasitic infection. Evolution 53: 426-434.

3. Otterstatter MC, Thomson JD (2006) Within-host dynamics of an intestinal pathogen of bumble bees. Parasitology 133: 749-761.

4. Schmid-Hempel P (1998) Parasites in Social Insects; Krebs JRaC-B, T.H., editor. Princeton, New Jersey: Princeton University Press.

5. Rodd FH, Plowright RC, Owen RE (1980) Mortality rates of adult bumble bee workers (Hymenoptera, Apidae). Canadian Journal of Zoology 58: 1718-1721.

6. Free JB (1955) The collection of food by bumblebees. Insectes Sociaux 2: 303-311.

7. Cartar RV (1992) Adjustment of foraging effort and task switching in energy manipulated wild bumblebee colonies. Animal Behaviour 44: 75-87.
