# Supplementary material for: Does Pathogen Spillover from Commercially Reared Bumble Bees Threaten Wild Pollinators?
Source: PLoS One. 2008 Jul 23;3(7):e2771. doi: 10.1371/journal.pone.0002771 (PMC2464710; doi:10.1371/journal.pone.0002771)
Supplement: Text S2 — Parameter estimates from the literature (0.07 MB DOC) [file pone.0002771.s002.doc]

## Supplementary Text S2

### Estimates of model parameters from the literature

*Death rates and birth rates (b*, *α, a)*

Rodd et al. [1] studied the mortality rate of wild bumble bees in eastern Canada; based on their life table, foragers (roughly, ages 10-26 days) have an average natural death rate of 0.183 d^-1^. We assumed that this value is a good estimate of disease-free mortality, *b*. The bees studied by Rodd et al. were almost certainly free of *C. bombi*, which was nearly absent (< 2% of wild *Bombus*) in eastern Canada around the time of their study [2].

Brown et al. [3] studied in the laboratory the mortality of bumble bees infected by *C. bombi*; they found that under food stress (nectar and pollen deprivation), infected bees had a 55.7% higher mortality rate than healthy bees. Most natural bumble bee colonies probably experience frequent food shortages, as well as other stresses (*e.g*., weather, predators, and pesticides) that may interact with parasitic infections; thus, it seems likely that wild bees harbouring *C. bombi* would experience, at the minimum, a similarly elevated mortality rate. This allows us to estimate disease-induced mortality, *α*, as 0.183 x 0.557 = 0.102 d^-1^.

The intrinsic, disease-free, growth rate of bumble bee populations is not well known. In our model, we assume that the net rate of increase, *a - b*, of the susceptible foraging population in the absence of *C. bombi* is positive, which reflects the rapid ‘growth phase’ that is typical of bumble colonies during the summer [4]. Given our value for *b*, we estimated a reasonable birth rate to be, *a* = 0.22 d^-1^; the resulting net rate of increase (0.22 – 0.183 = 0.037 d^-1^) produces wild bee densities in our simulations that are similar to those observed in nature [5]. We explore a range of values for the net rate of increase in our sensitivity analyses (Figure S1D).

*Transmission rate at flowers (ν)*

Few studies have attempted to measure rates of pathogen transmission in insects [reviewed by 6], and almost none consider transmission rates in bees [7,8]. However, Durrer and Schmid-Hempel [9] show that, in the laboratory, 20-40% of bumble bee workers become infected with *C. bombi* after foraging on flowers recently visited by an infected bee. Using this result, and our own data, we can estimate the transmission coefficient of *C. bombi* as $\nu=\frac{\mu}{P_{0}(e^{-\mu T}-1)}\ln(\frac{S_{T}}{S_{0}})$, where *µ* is our estimate of pathogen decay (= 12.98 d^-1^, see Results), and *S_T_* / *S_0_* is the fraction of initially susceptible bees that remained uninfected (36/51) after two hours of foraging (*T* = 0.083 d) in Durrer and Schmid-Hempel’s study. We used our estimate of pathogen deposition by infected bees (352 *C. bombi* per minute of foraging, see Results) to approximate the number of *C. bombi* initially present at flowers, *P_0_*, in Durrer and Schmid-Hempel’s study (352 cells/min x 180 min foraging = 63360 cells). The formula that we used to calculate *ν* was derived by D'Amico et al. [10] for estimating the transmission rates of an insect virus (nuclear polyhedrosis virus of the gypsy moth *Lymantria dispar*). Our calculated value for *ν* (1.08 x 10^-4^ m^2^ d^-1^) is within the range of transmission rates observed across a variety of insect-pathogen systems [6]. The sensitivity of our model to variation in *ν* is shown in Figure S1C.

*The diffusion coefficient (D)*

Adult winged insects can disperse a great distance from their initial location, e.g., *D* > 10^4^ m^2^ d^-1^ [11] and, in the case of solitary insects, this process is similar to diffusion [12]. Bumble bees, however, do not disperse like solitary insects: they make repeated trips between their colony and rewarding patches of flowers; hence, estimating a diffusion coefficient *D* is less straightforward than for solitary species. Nevertheless, it might be reasonable to assume that bees initially forage close to their nest and gradually expand their foraging range as they increasingly explore and acquire more information about their local habitat. In this case, we can assume that the distribution of individuals relative to their colony is approximated by a normal curve and estimate *D* as MSD/4*t*, where MSD is the (expected) mean squared displacement of bees at time *t* [11]. Most studies suggests that workers typically forage within ~500 m of their nest [13,14,15,16] and only occasionally venture beyond 1.5 km [17]. Assuming that an average worker disperses only as far as 1.5 km during its life [t = 25 days, from 1], yields *D* = 8.0 x 10^3^ m^2^ d^-1^. It is not known if infection alters a bee’s foraging distance; therefore, we assume the same value of *D* for healthy and infected workers. Infective *C. bombi* cells require bumble bees as vectors to move through the environment; thus we also use the same value of *D* for the dispersal of pathogen cells. We examine the predictions of our model across a wide range of values for *D* (Figure S1E).

# References

1. Rodd FH, Plowright RC, Owen RE (1980) Mortality rates of adult bumble bee workers (Hymenoptera, Apidae). Canadian Journal of Zoology 58: 1718-1721.

2. Liu HJ (1973) *Bombus* Latr. (Hymenoptera: Apidae) in Southern Ontario: Its role and factors affecting it [M.Sc. Thesis]. Guelph: Guelph University, Canada. 130 p.

3. Brown MJF, Loosli R, Schmid-Hempel P (2000) Condition-dependent expression of virulence in a trypanosome infecting bumblebees. Oikos 91: 421-427.

4. Schmid-Hempel P (1998) Parasites in Social Insects; Krebs JRaC-B, T.H., editor. Princeton, New Jersey: Princeton University Press.

5. Forup ML, Memmott J (2005) The relationship between the abundances of bumblebees and honeybees in a native habitat. Ecological Entomology 30: 47-57.

6. Fenton A, Fairbairn JP, Norman R, Hudson PJ (2002) Parasite transmission: reconciling theory and reality. Journal of Animal Ecology 71: 893-905.

7. Fries I, Lindstrom A, Korpela S (2006) Vertical transmission of American foulbrood (*Paenibacillus larvae*) in honey bees (*Apis mellifera*). Veterinary Microbiology 114: 269-274.

8. Chen YP, Pettis JS, Evans JD, Kramer M, Feldlaufer MF (2004) Transmission of Kashmir bee virus by the ectoparasitic mite *Varroa destructor*. Apidologie 35: 441-448.

9. Durrer S, Schmid-Hempel P (1994) Shared use of flowers leads to horizontal pathogen transmission. Proceedings of the Royal Society of London Series B 258: 299-302.

10. D'Amico V, Elkinton JS, Dwyer G, Burand JP, Buonaccorsi JP (1996) Virus transmission in gypsy moths is not a simple mass action process. Ecology 77: 201-206.

11. Kareiva PM (1983) Local movement in herbivorous insects: applying a passive diffusion model to mark-recapture field experiments. Oecologia 57: 322-327.

12. Dwyer G (1992) On the spatial spread of insect pathogens - theory and experiment. Ecology 73: 479-494.

13. Knight ME, Martin AP, Bishop S, Osborne JL, Hale RJ, et al. (2005) An interspecific comparison of foraging range and nest density of four bumblebee (*Bombus*) species. Molecular Ecology 14: 1811-1820.

14. Darvill B, Knight ME, Goulson D (2004) Use of genetic markers to quantify bumblebee foraging range and nest density. Oikos 107: 471-478.

15. Saville NM, Dramstad WE, Fry GLA, Corbet SA (1997) Bumblebee movement in a fragmented agricultural landscape. Agriculture Ecosystems & Environment 61: 145-154.

16. Dramstad WE (1996) Do bumblebees (Hymenoptera: Apidae) really forage close to their nests? Journal of Insect Behavior 9: 163-182.

17. Walther-Hellwig K, Frankl R (2000) Foraging distances of *Bombus muscorum*, *Bombus lapidarius*, and *Bombus terrestris* (Hymenoptera, Apidae). Journal of Insect Behavior 13: 239-246.
