# Supplementary material for: Does Pathogen Spillover from Commercially Reared Bumble Bees Threaten Wild Pollinators?
Source: PLoS One. 2008 Jul 23;3(7):e2771. doi: 10.1371/journal.pone.0002771 (PMC2464710; doi:10.1371/journal.pone.0002771)
Supplement: Table S1 — (0.04 MB DOC) [file pone.0002771.s005.doc]

**Table S1.** Summary of the four study sites in southern Ontario, Canada where we collected bumble bees during June-August, 2005.

| Study site | Collecting dates (# bees) | Collecting location | GPS coordinates | Distance to greenhouse |
| --- | --- | --- | --- | --- |
| Leamington | June 24-25 (72) August 19-20 (89) | vacant lot | 42 03.685 N - 82 36.121 W | 0.03 km |
|  |  | roadside ditch | 42 01.818 N - 82 34.984 W | 3.74 km |
|  |  | roadside ditch | 42 01.572 N - 82 33.417 W | 5.30 km |
|  |  | old field | 42 01.027 N - 82 33.461 W | 6.05 km |
| Exeter | June 21-22 (82) July 25-27 (136) August 16-18 (164) | service road | 43 22.318 N - 81 26.477 W | 0.03 km |
|  |  | roadside ditch | 43 22.039 N - 81 26.318 W | 0.17 km |
|  |  | roadside ditch | 43 22.335 N - 81 26.074 W | 0.65 km |
|  |  | old field | 43 23.380 N - 81 26.305 W | 2.40 km |
| Beamsville | July 28 (41) | vacant lot/roadside ditch | 43 11.534 N - 79 24.337 W | 0.03 km |
| Thamesville | July 23 (32) | old field | 42 33.560 N - 81 59.360 W | > 50.00 km |
